# Prognostication from Kidney Transplant Biopsies: 90 Years of Dynamic Alien Landscape Forward and Back

**DOI:** 10.34067/KID.0000000000000534

**Published:** 2024-09-26

**Authors:** Kim Solez

**Affiliations:** Department of Laboratory Medicine and Pathology, University of Alberta, Edmonton, Alberta, Canada

**Keywords:** chronic kidney failure, glomerulosclerosis, kidney biopsy, kidney transplantation, rejection, stem cell, transplant pathology, transplantation, artificial intelligence, tubulointerstitial disease

For almost everyone, scarred kidney histopathology is at some point a surprisingly alien landscape, appearing to be much more static and adynamic than it is. This was first brought home to me in 1981 when I was visiting the 5500-year-old Poskaer Stenhus burial mound in Denmark with nearby Aarhus University pathology chief Steen Olsen. Aarhus is the second largest city in Denmark, and Steen had previously been the Dean of Medicine at the University of Aarhus and was one of my mentors. He joked that sclerotic glomeruli seen on kidney biopsy were as unchanging as the ancient burial stones we were encountering at Poskaer Stenhus. I told him about the 1934 autopsy study by Moritz and Hayman^1^ showing that sclerotic glomeruli of CKD disappear into the interstitial scar tissue. He was surprised and resistant to the idea at first, but then came around when presented with the details from the paper. Since then, I have had numerous similar conversations with other renal pathologists, students, and clinicians young and old over many decades.

The recent paper by Denic *et al.*^2^ presents yet another surprise: Contraction of interstitial fibrosis/tubular atrophy (IFTA) may cause %IFTA to under-represent the severity of nephron loss. These authors found that counting IFTA foci density in kidney transplant biopsies provides much more accurate prognostic predictions because of this shrinkage of scars. The study found that morphometric measurements of %IFTA and IFTA foci density are more predictive of allograft failure than the traditional Banff ci score. Unlike the Banff ci score, which did not correlate significantly with allograft failure after adjusting for other variables, the morphometric measures retained their predictive power in various statistical models. This suggests that measuring %IFTA and IFTA foci density could significantly enhance the monitoring and management of kidney transplant recipients. These measures could serve as important surrogate end points for clinical trials, potentially leading to earlier interventions to preserve allograft function. If validated by a second independent research group, one could justify adding this parameter to routine diagnostic interpretation of native and allograft kidney biopsies and inclusion in the current diagnostic pathology standard of care and Banff classification guidelines.

One might expect these relationships between IFTA foci density and prognosis to be species specific, so one would have to work out new details when dealing with xenografts/pig-to-human transplants or with regenerative medicine constructs that involve reseeding of pig scaffolds with human cells. That is important because the recent prediction of having “an end to the organ shortage”^3^ or “unlimited organs”^4^ in the next 10–15 years cannot be accomplished with allografts alone, which currently provide only 5%–10% of the organs needed, but will probably require the addition of a combination of xenografts, regenerative medicine constructs, and bioartificial organs.

These changes will likely be speeded up, at least for now, by a decision made by the US Supreme Court on June 28, 2024, removing from regulatory agencies the right to have the final word and giving that right back to the courts by ending what is known as “Chevron Deference,” which embodies the regulatory understanding that has existed for the past 40 years.^5,6^ The Natural Resources Defense Council has suggested^7^ that this decision will bring chaos, but with proper planning this need not be the case, at least in the transplantation arena. One could imagine a coordinated effort to expand xenotransplantation to patients on the waitlist for allografts but where the wait for an organ will be quite prolonged and the quality of life on dialysis is quite low, and then from there gradually expanding the field of transplantation ten-fold, where ten-fold more organs are produced, ten-fold more people are involved, and ten-fold more investment takes place. An energetic public relations campaign would be required to recruit new young people to the field. Clinical trials were initially expected in 2022, but now we will likely have them in 2025. Exactly how all this will work is unknown, and so we will have to accept some additional surprises.

American Pie excerpt (Don McLean, 1971)^8^

“We all got up to dance

Oh, but we never got the chance

’Cause the players tried to take the field

The marching band refused to yield

Do you recall what was revealed

The day the music died?”

It is useful to consider the 73-year history of the human kidney biopsy and the 33-year history of the existing Banff Classification.^9^ Kidney biopsy was introduced in 1951 by Poul Iversen and Claus Brun, Peter Medawar described immunologic tolerance in 1953, kidney transplantation was introduced in 1954 by John P. Merrill and Joseph Murray, the first edition of Robert Heptinstall's Pathology of the Kidney was published in 1966, and he became the President of the American Society of Nephrology in 1972, the year I joined him as a trainee. The Renal Pathology Club was formed in 1977 and became the Renal Pathology Society in 1993. US Food and Drug Administration approval of cyclosporin as an immunosuppressive agent occurred in 1983, and tubulitis was identified as a specific lesion of rejection in 1986. The Banff Classification was founded in 1991. In 1997, the Banff and Cooperative Clinical Trials in Transplantation classifications merged. Antibody-mediated rejection was added in 2001. Peritubular capillary microvascular inflammation scoring and total i scoring (in both scarred and unscarred renal parenchyma) were added in 2007. Banff Working Groups were started in 2009. C4d-negative antibody-mediated rejection and antibody-mediated intimal arteritis (the v lesion) were introduced in 2013. Precision medicine and regenerative medicine approaches were described in 2015. A reference guide was published in 2018. The Banff Digital Pathology Working Group was established in 2019. The Banff Human Organ Transplant gene panel was also established in 2019. In 2024, iBox is poised to become the surrogate end point for late kidney transplant failure.^9^

**Figure 1:**
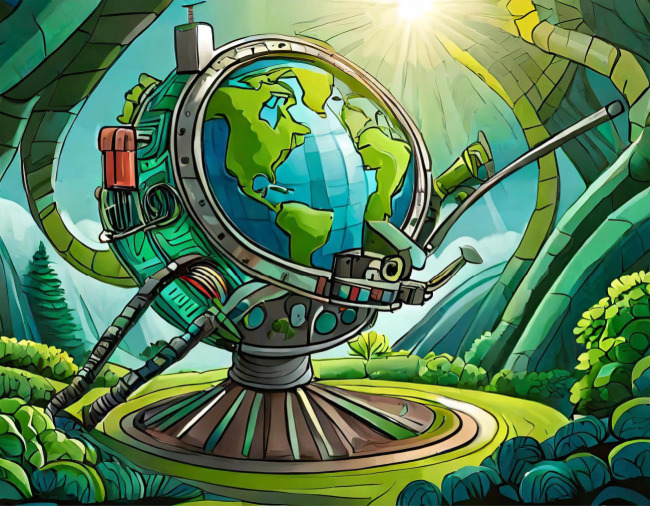
Adobe Firefly artificial intelligence image of a completely automated world optimized for human flourishing.

In the very long run, however, having gone back 90 years to 1934,^2^ if we project 90 years forward into the future of a solved world^10^ of 2114 where artificial intelligence (AI) is doing almost everything, there is another big surprise because by that time it is likely that every element of transplantation will be automated (Figure 1), except the patients, so machines and AI can do the clinical assessment, the surgery, and even the pathology. The only people in the process still with jobs will be the patients, and they will likely be happy because the whole process will be optimized for them by superhuman AI. There will indeed be multiple Banff classifications^9^ applied to the various solutions to the organ shortage, but mainly updated and paid attention to by machine intelligences. So, here we have a story with a happy ending, but probably not the one you were expecting, and with an eventual substantial nonhuman audience.
